# Laryngeal Electromyography as a Predictive Factor in the Evolution of Unilateral Recurrent Paralysis Post-Thyroidectomy

**DOI:** 10.3390/jcm14041047

**Published:** 2025-02-07

**Authors:** Shirley Tarabichi, Codrut Sarafoleanu

**Affiliations:** 1 Faculty of Medicine, “Carol Davila” University of Medicine and Pharmacy, 20021 Bucharest, Romania; 2 ENT&HNS Department, “Sf. Maria” Clinical Hospital, 011172 Bucharest, Romania

**Keywords:** laryngeal electromyography, dysphonia, thyroidectomy, vocal fold paresis, laryngeal recurrent nerve, recovery, prognosis

## Abstract

**Background**: Dysphonia, a common symptom after thyroid surgery, is most often caused by damage to the recurrent laryngeal nerve. Laryngeal electromyography (LEMG) is used as a qualitative diagnostic tool to distinguish neurological etiology from other causes of dysphonia. The purpose of this study is to establish the value of LEMG as a predictor factor in the recovery of unilateral recurrent paralysis post-thyroidectomy. **Methods**: This study included 11 patients with unilateral vocal fold palsy (UVFP) evidenced on the videostrobolaryngoscopy (VSL) after thyroidectomy. Electrical activity of thyroarytenoid (TA) muscles of the patients included in the study was recorded through LEMG and the prognosis of the lesions was classified as excellent, fair, or poor based on the presence of spontaneous activity and motor unit recruitment. **Results**: LEMG at the first clinic visit showed an excellent prognosis in three of the cases, a fair prognosis in three of the cases, and five of them indicated a poor prognosis. At 6 months after the first LEMG, patients with a poor prognosis were unchanged and showed no LEMG improvement. Those with an excellent prognosis showed an increased recruitment response, and LEMG was normal. In one patient with a fair prognosis and minimal spontaneous activity, LEMG recruitment decreased during reevaluation. The other two fair-prognosis patients had a normal LEMG. **Conclusions**: A correlation was found between LEMG findings and functional recovery of the vocal cords, demonstrating that the presence of spontaneous activity represents a negative prognostic factor. However, due to limited patient cohorts, the sensitivity of the LEMG as a prognostic tool in the functional recovery of the larynx is not yet established and requires further research.

## 1. Introduction

Dysphonia is frequently reported by patients as a new symptom after a thyroidectomy. Even though the etiology of dysphonia is variated, paralysis of the vocal cord, caused by injury of the recurrent laryngeal nerve is considered to be the main cause. This paralysis often leads to inadequate closure of the glottis, which produces dysphonia, characterized by vocal weakness, lower voice amplitude, and volume [1].

The incidence of nerve damage has not been commonly reported in relation to the underlying condition, the affected nerve, or both. Moreover, a significant number of studies were retrospective, including surgeries performed over an extended period of time by various surgeons [2].

In the literature, voice changes post-thyroidectomy represent a common concern, reported by 25% to 90% of the patients [3,4,5,6,7]. The incidence of irreversible nerve injury following thyroid surgery varies from 0% to 5.8%, but the rates increase between 13% and 30% in patients with thyroid cancer [8].

Additionally, voice changes after thyroidectomy can occur even in the absence of noticeable nerve damage. The literature highlights many alternative pathophysiological mechanisms that are potentially responsible for the hoarseness and do not involve injury to the recurrent laryngeal nerve. Among these, there are post-surgical fibrosis, arytenoid fixation, venous congestion secondary to intraoperative vascular ligation, lymphatic disruption leading to vocal fold edema, and the compressive effects of the intubation balloon used during general anesthesia [9].

Patients with dysphonia experience social isolation and limited conversational involvement, which affect their social and professional lives. Consequently, these patients often experience depression and anxiety, accompanied by decreased self-confidence. Dysphonia is a significant impediment to occupational performance for professional voice users, including teachers, public speakers, and singers. The inability to maintain vocal performance often results in diminished productivity, absences from work, or, in extreme instances, the necessity to leave the profession [10]. Research indicates that the effect of dysphonia on quality of life is similar to that of chronic diseases including asthma and chronic obstructive pulmonary disease [11]. Other research has concluded that moderate dysphonia is analogous to monocular blindness regarding its impact on quality of life [12]. Given the importance of dysphonia on quality of life, early diagnosis and prognosis assessment are helpful for guiding the choice of subsequent treatment methods in order to reduce its impact on quality of life.

In order to analyze voice quality, dysphonia analysis typically uses a multidimensional approach that integrates both subjective and objective evaluation methods. Subjective instruments such as the Voice Handicap Index (VHI) [13] and voice-related quality of life (VRQOL) [14] highlight the patient’s individual experience, while objective instruments like acoustic voice quality index (AVQI) have the ability to provide quantifiable accurate data through the use of Pratt software (Vocal Outcome Following Thyroidectomy for Differentiated Thyroid Carcinoma). Health-related quality of life tools such as the EuroQOL 5-Dimension Questionnaire (EQ-5D) [15] and Short Form 36 Health Survey (SF-36) [16] allow clinicians to evaluate the broader health implications of dysphonia, particularly in cases where the condition significantly affects the patient’s mental or physical health.

In diagnosing neurolaryngological disorders, it is widely acknowledged that laryngeal electromyography serves predominantly as a qualitative diagnostic tool [17].

For this study, to distinguish a neurological etiology from other causes of laryngeal complications after thyroidectomy, we used laryngeal electromyography (LEMG) for accurate diagnosis.

This retrospective study aims to determine the value of LEMG as a predictive factor in the recovery of unilateral recurrent paralysis post-thyroidectomy.

The data obtained through LEMG play a crucial role in identifying the nature of nerve damage and predicting both the timeline and the outcome of the recovery process.

## 2. Materials and Methods

A total of 18 patients with laryngeal complications after thyroidectomy presented to our clinic between 2021 and 2023. All patients signed an informed consent form, and the local ethics committee approved the protocol before starting the study.

In each patient, a complete ENT examination was performed at the time of presentation, including videostrobolaryngoscopy (VLS). The patients were also evaluated using the VHI, which allowed us to quantify the subjective experiences of patients. This provided a more comprehensive view of how dysphonia affects the patient’s daily life and general well-being.

Based on VLS images, information about vocal fold vibrations and glottal closure—complete glottal closure, incomplete closure of the membranous portion of the vocal fold, and incomplete closure of the entire glottis—was provided.

Vocal fold paralysis was defined as the absence of movement of one or both vocal folds during phonation. Paresis of the vocal cord was defined as decreased movement of one or both vocal folds during phonation.

The inclusion criteria for the study were defined as follows:

1. Patients aged 18 to 65 years;
2. Diagnosed with unilateral vocal fold palsy (UVFP) as evidenced by videostroboscopy (VSL) after thyroidectomy;
3. The time between the injury and presentation to the clinic was less than 6 months.

The exclusion criteria for the study were defined as follows:

1. Patients with other causes of laryngeal complications, such as arytenoid fixation;
2. A history of
  - Vocal fold paralysis;
  - Voice or laryngeal diseases requiring therapy;
  - Pulmonary disease;
  - Previous neck surgery;
  - Invasion of the RLN or vagus nerve identified on the CT scan before thyroidectomy;
3. Patients who failed to appear for reevaluation.

This ensures that the study concentrates only on cases of UVFP directly caused by thyroidectomy during a specific period of time.

Overall, 11 patients were included in the study. There was 1 male and 10 females in the study, with an age range of 22 to 62 years and a median age of 42 years. All 11 patients were diagnosed with benign thyroid lesions through histological exam.

## 3. LEMG

The electrical activity of thyroarytenoid (TA) muscles of the patients included in the study was recorded through LEMG (Natus viking system: EMG/EP 8 channels Software Viking EDX, 3.0.6). A 45 × 0.45 mm concentric needle electrode attached to a Neurosoft EMG machine was used to collect LEMG signals. The ground electrode was attached to the patient’s hand.

For the examination, the patients were seated with their necks slightly extended. After sterilizing the neck skin with alcohol, the needle electrode was inserted through the cricothyroid membrane along the midline. After the needle penetrated the cricothyroid membrane, it was angled laterally (30 degrees) and upwards (15 degrees). The electrode’s positioning was confirmed by observing an increase in electrical impulses on the monitor when the participant pronounced the vowel “i”. Patients were then asked to articulate a series of sounds of the vowel ‘a’ at a moderate volume.

We analyzed the LEMG signals from the TA muscles and classified the prognosis of the lesions, using the following labels: excellent, fair, and poor (Table 1).

Spontaneous activity (fibrillations, positive waves, fasciculation, and high-frequency complexes) registered during rest and the recruitment of motor unit potentials (laryngeal recruitment) registered during the volitional activity were analyzed for the classification. If the TA muscle of the affected vocal fold had normal to nearly normal recruitment during phonation and absence of spontaneous activity, the prognosis was defined to be excellent (LEMG-negative finding). The prognosis was defined to be poor (LEMG-positive finding) if the affected vocal fold had low to very low recruitment and the presence of spontaneous activity, and mild if the recruitment was low and there was no spontaneous activity.

## 4. Statistical Analysis

Statistical analysis was performed using commercially available statistical software (SPSS 29.0). The chi-square test was used to evaluate the relationship between LEMG parameters, prognosis, and functional recovery. A value of *p* < 0.05 was considered significant.

## 5. Results

LEMG was performed on 11 patients (10 females and 1 male) with a mean age of 42 years (range: 22 to 62 years) who complained of vocal changes after the thyroidectomy surgery and met the inclusion criteria. Also, all patients had dysmobility on the left side. The distribution of the patients included in our study is represented in Table 2.

A total of three patients presented with a nearly normal recruitment LEMG pattern for the TA on both sides (recruitment decreased very little, by 10%, and no spontaneous activity was registered)—LEMG-negative findings. For these patients, the prognosis was defined as excellent.

In two patients, decreased recruitment was observed, but no spontaneous activity was noted and in one patient, minimal spontaneous activity and normal recruitment were registered. Based on this, we classified these patients as having a fair prognosis.

For five of the patients, the LEMG of the TA muscle revealed diminished motor unit recruitment and the presence of some spontaneous activity as fibrillation potentials. We consider these patients to have LEMG-positive findings and classify them as having a poor prognosis.

The LEMG performed at the first visit to the clinic showed excellent prognosis in 27% of the cases (three patients). A fair prognosis was also observed in 27% of the cases (three patients). A poor prognosis was registered in 45% of the cases (five patients) (Table 3).

## 6. Follow-Up

Six months after the first evaluation, the patients returned to the clinic for a full reevaluation. During this time, all patients underwent voice therapy.

Vocal symptoms (dysphonia) persisted in only six of the eleven patients (54%) who had voice complaints. Four patients (36%) declared that their voice seemed to be normal with a VHI of <30. One patient (9%) affirmed that there was an improvement in their voice, also with a VHI of <30. No patient developed new symptoms of dysphonia or had an increase in their VHI (Table 4).

All the patients underwent a VLS reexamination to evaluate the recovery of vocal fold mobility. Clinically, five patients (45%) regained normal mobility of the vocal folds, while six patients (54%) showed no clinical recovery (Table 5). If recurrent nerve paralysis persists for more than one year after the injury, it is considered permanent.

Each patient was reevaluated with LEMG. Patients who had positive findings on the first LEMG (five of eleven patients—45%) were found to have remained the same and no improvement was seen on the new LEMG. All three patients (27%) with LEMG-negative findings and excellent prognosis experienced an increase in the recruitment response, and the LEMG was normal. In the case of the one patient with the presence of minimal spontaneous activity, which was classified as having a fair prognosis, the reevaluation showed a decrease in recruitment on the LEMG. In the other two patients with a fair prognosis, a slight improvement was observed, with an average of 15% increase in recruitment response, resulting in a normal LEMG. There was no evidence of abnormal LEMG in any of the patients who had improved.

The distribution of patients based on gender, initial and second LEMG findings, and their clinical recovery outcomes after six months is shown in Table 6.

We considered that the presence of spontaneous activity on the LEMG is statistically significant (*p* < 0.01) in predicting the absence of functional recovery of the vocal cords.

For this reason, we emphasize that LEMG has a high specificity for predicting the functional recovery of the vocal cords. Moreover, we consider the presence of spontaneous activity the most significant factor in predicting the absence of recovery.

## 7. Discussion

Numerous studies have been conducted in the literature to investigate the difficulties that can arise following thyroid surgery [18,19,20,21]. In the first weeks after surgery, between 25% and 90% of patients experience abnormal voice, and between 11% and 15% of them experience dysphonia that lasts for six months after surgery [18,21].

Iatrogenic injuries that occur during surgery and malignancy are the primary causes of unilateral vocal fold paralysis, as stated in the research conducted by Rosenthal and colleagues [22]. Thyroidectomy continues to be the main surgical procedure associated with iatrogenic UVFP, according to many series of studies that have indicated an increase in the incidence of iatrogenic injuries [23,24].

LEMG has been considered predominantly a qualitative method of investigation [17]. However, it has been demonstrated that LEMG has a high positive predictive value (PPV) in predicting the long-term prognosis of patients with a poor prognosis. Furthermore, it is commonly used to predict recovery, regardless of the etiology responsible for vocal fold paresis [25,26,27,28].

In cases of postoperative vocal fold paresis following thyroid surgery, the prognostic information obtained from LEMG can be helpful in identifying cases that may require future interventions. These interventions may include surgical or pharmacological reinnervation therapies, as well as surgery for vocal fold medialization [29].

The aim of this study is to evaluate the predictive value of LEMG in determining the recovery of laryngeal function in cases of UVFP after thyroidectomy.

Multiple studies have used LEMG data, especially spontaneous activity at rest and recruitment during phonation, to determine the prognosis of neurological lesions in cases of UVFP. The evaluation also includes determining sensitivity and specificity values [4,5,6,7,8].

The meta-analysis by Rickert et al. classified neural lesion severity as low or high grade [25].

In the present study, the prognostic criteria were also based on the presence of spontaneous activity at rest and recruitment during phonation. The presence of spontaneous activity and decreased recruitment were considered indicative of a high-grade lesion with a poor recovery prognosis. The absence of spontaneous activity and quite normal recruitment was considered indicative of a low-grade lesion with an excellent prognosis. Additionally, we found two patients with the absence of spontaneous activity but with reduced recruitment, and one patient with normal recruitment but the presence of spontaneous activity. We considered these modifications suggestive of a moderate lesion with the hope of recovery.

In order to obtain the correct LEMG data after thyroid surgery, it is essential to take into consideration the time of the examination.

Denervation activity, which is a sign of axonotmesis and a negative prognosis, usually becomes evident three weeks after the injury to the recurrent laryngeal nerve (RLN) and continues until the process of reinnervation is completed [30]. The reinnervation of the intrinsic laryngeal muscles is likely to occur quickly due to the RLN’s strong ability to regenerate and the additional reinnervation from nearby, undamaged nerve fibers [31,32].

The EMG is typically performed three weeks after the onset of symptoms. This period is established by neurologists. After three weeks, the nature and degree of the lesion can be determined. However, after two months, the presence of the reinnervation can be observed, which is one of the factors that is truly associated with the prognosis [33].

A minimum of two months must pass between the onset of symptoms and the laryngeal electromyography, as demonstrated by Wang et al. [28], in order to improve both the PPV and the specificity.

Hirano et al. [34] suggest performing the LEMG within 6 weeks of symptom onset and excluding patients in whom more than 6 months have passed, stating that few patients have recovered after that interval.

Parnes et al. [35] claim that the time interval from two weeks to six months is ideal for performing LEMG.

In the present study, the interval for performing LEMG was two to six months after the onset of symptoms.

In the literature, no specific timeframe has been established for the period preceding the second LEMG. However, it is important to adapt this interval to the particular case of vocal fold palsy and its associated symptoms.

We consider the most favorable time to perform the second LEMG to be six months following the initial one.

In 2012, Rickert and colleagues [25] published a meta-analysis of studies that reported the results and clinical outcomes of LEMG. The studies included a total of 503 patients and showed a positive predictive value (PPV) of 90.9% and a negative predictive value (NPV) of 55.6%. In 2014, Wang and colleagues [27] conducted a prospective study to predict the long-term prognosis of UVFP, which included 85 patients. They reported a PPV of 93% and an NPV of 40% [27].

In our study, we demonstrated that the presence of spontaneous activity on LEMG has a high PPV for a negative outcome, emphasizing that it is an effective method for predicting the absence of functional recovery of the vocal cords.

Within the literature, there is variation in the prognostic criteria across different studies. However, many of these criteria essentially correspond with Seddon’s classification of nerve injury. According to Seddon’s classification, the absence of voluntary motor units and the presence of spontaneous activity, such as fibrillation potentials, as indicators of axonotmesis or neurotmesis, suggest a negative prognosis. On the other hand, the presence of voluntary motor unit potentials and recruitment without spontaneous activity indicates neuropraxia, which is considered to have a more favorable prognosis [23].

The presence of spontaneous activity and the absence or diminished recruitment of motor unit potentials are two characteristics that have been suggested by a number of studies to be indicative of a poor prognosis [18,19,21,22,24,35,36]. Other studies required a strict absence of motor unit potentials to define a poor prognosis [34,37].

As mentioned earlier, in our study, the data we collected include two key parameters: the presence of spontaneous activity and the recruitment of motor unit potentials.

## 8. Conclusions

In this study, we emphasize that the absence of spontaneous activity is a positive prognostic sign, while its presence, even if minimal, is an indicator associated with a poor prognosis. On the other hand, in patients in whom LEMG showed a decrease in recruitment but no spontaneous activity, we observed not only improvement in their symptomatology and on VLS, but also increased recruitment on the LEMG. This suggests that a decrease in recruitment without the presence of spontaneous activity cannot be considered a negative prognostic factor.

However, the sensitivity of LEMG parameters as a prognostic method remains variable due to the limited number of patient cohorts reported in the literature. Also, further research with a larger number of patients is needed to highlight the sensitivity and improve the predictive accuracy of LEMG parameters in clinical practice.

## Figures and Tables

**Table 1:** Prognosis of recovery based on LEMG.

Prognosis	Recruitment of TA Muscle	Spontaneous Activity	Lemg Finding
Excellent	Normal to nearly normal during phonation	Absent	Negative
Fair	Low recruitment during phonation	Absent	Negative
Poor	Low to very low recruitment during phonation	Present	Positive

**Table 2:** Distribution of the patients based on gender, age, and side of the lesion.

**Number of cases**	11
**Sex (male/female)**	1/10
**Mean age**	42 years
**Side of the lesion (left/right)**	11/0

**Table 3:** LEMG prognosis.

Prognosis		Percent (%)	Valid Percent (%)
Poor	5	45.5	45.5
Fair	3	27.3	27.3
Excellent	3	27.3	27.3
Total	11	100.0	100.0

**Table 4:** Distribution of dysphonia outcomes among patients.

Dysphonia		Percent (%)	Valid Percent %
Persisted	6	54.5	54.5
Improvement	1	9.1	9.1
Absent	4	36.4	36.4
Total	11	100.0	100.0

**Table 5:** Distribution of clinical recovery among patients.

Clinical Recovery		Percent (%)	Valid Percent (%)
No	6	54.5	54.5
Yes	5	45.5	45.5
Total	11	100.0	100.0

**Table 6:** Distribution of patients by gender, LEMG prognosis, and clinical outcomes at the six-month follow-up.

Gender	At Presentation			Reevaluation After 6 Months			
	First LEMG		Prognosis	Second LEMG (After 6 Months)		Clinical Recovery (VLS)	Dysphonia
	Spontaneous Activity	Recruitment of Motor Unit Potentials		Spontaneous Activity	Recruitment of Motor Unit Potentials		
W	Yes	decreased	Poor	Yes	decreased	No	Persisted
W	Yes	decreased	Poor	Yes	decreased	No	Persisted
W	Yes	decreased	Poor	Yes	decreased	No	Persisted
W	Yes	decreased	Poor	Yes	decreased	No	Persisted
W	Yes	decreased	Poor	Yes	decreased	No	Persisted
W	No	decreased	Fair	No	Normal	Yes	Absence
M	No	decreased	Fair	No	Normal	Yes	Improvement
W	Yes (minimal)	Normal	Fair	Yes	Decreased	No	Persisted
W	No	Slightly decreased	Excellent	No	normal	Yes	Absence
W	No	Slightly decreased	Excellent	No	Normal	Yes	Absence
W	No	Slightly decreased	Excellent	No	Normal	Yes	Absence

## Data Availability

The raw data supporting the conclusions of this article will be made available by the authors on request.
